# The practice of tracheostomy decannulation—a systematic review

**DOI:** 10.1186/s40560-017-0234-z

**Published:** 2017-06-20

**Authors:** Ratender Kumar Singh, Sai Saran, Arvind K. Baronia

**Affiliations:** Department of Critical Care Medicine, Sanjay Gandhi Post Graduate Institute of Medical Sciences (SGPGIMS), Raebareli Road, Lucknow, 226014 Uttar Pradesh India

**Keywords:** Tracheostomy, Decannulation, Weaning

## Abstract

Decannulation is an essential step towards liberating tracheostomized patients from mechanical ventilation. However, despite its perceived importance, there is no universally accepted protocol for this vital transition. Presence of an intact sensorium coordinated swallowing and protective coughing are often the minimum requirements for a successful decannulation. Objective criteria for each of these may help better the clinical judgement of decannulation. In this systematic review on decannulation, we focus attention to this important aspect of tracheostomy care.

**Electronic supplementary material:**

The online version of this article (doi:10.1186/s40560-017-0234-z) contains supplementary material, which is available to authorized users.

## Background

Tracheostomy is a common procedure in patients requiring prolonged mechanical ventilation (MV) and airway protection in intensive care unit (ICU) [1]. The process of weaning from tracheostomy to maintenance of spontaneous respiration and/or airway protection is termed “decannulation”. This apparently simple step requires a near perfect coordination of brain, swallowing, coughing, phonation and respiratory muscles [2]. However, multifactorial aberrations in this complex interplay can result in its failure. Moreover, inappropriate assessment of the above factors increases the risk of aspiration during and after the decannulation process. Old age, obesity, poor neurological status, sepsis and tenacious secretions are the predominant reasons of failed decannulation [3].

Inability to speak with tracheostomy tube (TT) in situ results in significant anxiety and depression amongst patients [4]. More often than not, the process of decannulation is slow and prolonged leading to increased ICU stay, nosocomial infections and costs [5]. Provision of optimal tracheostomy care can help discharge these patients with TT in situ to ward, high dependency unit (HDU) and/or home. Repeat assessment and decannulation can then be performed during follow-up visits. Several studies have emphasized the importance of decannulation within the ICU due to better and focused care compared to HDU or ward [6, 7].

Inspite of the relevance and importance of decannulation, there is no universally accepted protocol for its performance. Variability in existing algorithms [8], non-randomized study design [9] and ambiguity in the screening, technique and monitoring of decannulation limits our understanding in this important area of care. In order to better understand the various practices of tracheostomy decannulation, we performed the present systematic review of the process of decannulation.

## Material and methods

### Criteria for including studies

Case series, case–control, prospective, retrospective, randomized or non-randomized studies or surveys dealing with the process of decannulation were all included in this systematic review.

### Patients

Adult patients aged above 18 years and admitted in ward, operation theater, ICU or HDU were included.

### Interventions

Patients with surgical or percutaneous dilatational tracheostomy who were subjected to the process of decannulation during weaning from MV were included.

### Outcome measures

Primary outcome measure assessed was success of weaning defined by a period of spontaneous breathing without having to resort to non-invasive ventilation (NIV) support or re-insertion of TT.

### Identification of studies

Two independent reviewers searched the electronic database PubMed using mesh words “Tracheostomy”, “Decannulation” and “Decannulation process” as title for the intervening period from 1995 to 2016 for identification of studies. The third independent reviewer then screened the two lists, removed the duplicates, and then searched for the abstracts which fulfilled the inclusion criteria. Full texts of the selected abstracts were then retrieved. Studies which further detailed the aspects of the “process of decannulation” were included. References of the included studies were further searched for any additional relevant studies not identified through our former search.

### Study selection

Only studies wherein full texts were available were finally included. In case full-text article was not available for a selected study, the institutes e-library using “ERMED consortium” and/or “Clinical key” were used for free access to journals. In the event free access to full text was still not available then the authors were contacted directly for copies. English language and full-text restriction were used for inclusion of relevant studies.

### Data extraction

Author (s), year of publication, country, type of study (observational, cohort, case–control, randomized and or survey), characteristics of patients, nature and severity of illness, site of care (ward, OT, HDU or ICU), method by which tracheostomy was performed [surgical or percutaneous dilatational (PCD)], length of MV prior to decannulation, criteria and method of decannulation used, outcomes in terms of success or failure of decannulation, definition of failed decannulation and limitations of study were all assessed. For completeness of data, any missing information was retrieved by directly contacting the respective authors.

### Quality assessment

The methodological quality of randomized controlled studies was assessed by Jadad scale while non-randomized studies were assessed using the “Q-Coh” tool for cohort studies in systematic reviews and meta-analyses [10]. Q-Coh is a 9-point tool which incorporates the attributes of design, representativeness, and comparability of groups, exposure measures, and maintenance of comparability, outcome measures, attrition, statistical analysis and the overall assessment of each study.

## Results

**Fig. 1:**
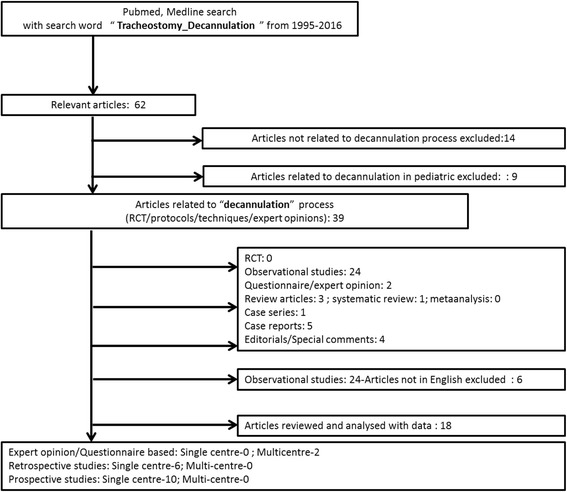
Flow diagram of selection of studies

Our PubMed database search yielded 62 articles published between January 1995 and December 2016. Fourteen articles were excluded as they were not related to the process of decannulation. Another 9 articles from paediatrics were also excluded. The remaining 39 articles included 24 observational studies, 5 case series, 1 case report, 4 editorials and special comments, 2 questionnaires or expert opinions and 1 systematic review. There was no randomized controlled study. Six studies each were further excluded owing to non-availability of data and use of language other than English. The final number of full-text studies thus included in our analysis was 18. The step-wise selection of studies along with reasons for exclusion was as enumerated in Fig. 1. After analyzing the selected studies, we decided to perform a systematic and critical review of the existing studies on decannulation due to lack of statistical requirements for a meta-analysis.

The detailed characteristics of the finally included 18 studies were as depicted in the Tables 1 and 2. There were 10 prospective [8, 9, 11–18], 6 retrospective studies [4, 19–23], and 2 questionnaire-based surveys [24, 25]. There was no randomized controlled study. The 16 prospective and retrospective studies were all single centre, while both surveys were multicentre. Except one study from India [9], all were from the developed world. In all, a total of 3977 patients with age varying between 24 and 85 years were included in these studies. The largest numbers of patients included were 981 in the prospective study by Choate et al. in 2009 from Australia [14].

**Table 1:** Characteristics of included studies

Author	Country	Year of publication	Type of study	Category of patients	Number of patients	Age (years)	Duration of MV (days) prior to decannulation	Surgical/PCT	Inclusion criteria	Exclusion criteria
Graves A et al. [11]	USA	1995	Prospective single centre	Chronic neurological illness	20	58	44–54	NA	1. Ventilation for 4 weeks 2. Successfully weaned off for 48 h 3. Minute ventilation <10 L/min 4. RR <12 5. SaO_2_ >90% (0.4 FiO2)	NA
Bach et al. [12]	USA	1996	Prospective single centre	Chronic neurological illness	49	24–62	287–2224	NA	Medically stable Afebrile N WBC counts Not receiving IV antibiotics Cognitively intact Not on narcotics/sedation Peak cough flow (PCF) PaO_2_ >60 mmHg SaO2 >92% N PaCO_2_ ± ventilation and use of manually/mechanically assisted coughing	NA
Ceriana et al. [8]	Italy	2003	Prospective single centre	Non-respiratory, 58% Chronic respiratory failure, 40%	72	59–77	8–72	Mainly surgical	Clinical stability Absence of psychiatric disorders Effective cough (MEP ≥40 cmH_2_O) PaCO_2_ <60 mmHg Adequate swallowing (evaluated by gag reflex or blue dye test) No tracheal stenosis endoscopically Spontaneous breathing ≥5 days.	NA
Leung et al. [19]	Australia	2003	Retrospective single centre	Respiratory, 35% Neurological, 35% Trauma, 17%	100	65	25	Surgical, 47 PCT, 53	Not mentioned	NA
Tobin et al. [13]	Australia	2008	Prospective single centre	Medical, 40% Surgical, 14% Cardiothoracic, 25% Neurosurgical, 23%	280	61.8	NA However, 58 pts on prolonged MV	Surgical, 15 PCT, 85	Tolerate capping >24 h Cough effective (No need of suctioning). Speech (with Passey–Muir valve).	NA
Stelfox et al. [24]	USA	2008	Questionnaire-based study Multicentre (118 centres)	Stroke, 166(24) Respiratory failure, 159(23) Trauma, 168(24) Abdominal aortic aneurysm, 182(27)	675 case scenarios	NA	NA However, majority physicians were from acute care.	NA	NA	NA
Choate et al. [14]	Australia	2009	Prospective single centre	Medical, 190 Surgical, 362 Trauma, 429	981	35–77	9–25	Surgical, 77% PCT, 23%	Weaned from ventilator Normal gag reflex Effective cough Reason for TT resolved Ability to swallow own secretions SaO_2_ >90%	Tracheotomies by ENT surgeons were excluded
O Connor et al. [4]	USA	2009	Retrospective single centre	Pneumonia, 25 Aspiration pneumonia or pneumonitis, 25 AECOPD, 25 Septic shock, 25	135	74(36–91)	45	NA	NA	NA
Chan LYY et al. [15]	Hong Kong	2010	Prospective single centre	Neurosurgical patients	32	49–80	13.32	NA	Hemodynamically stable Body temp <38 °C Inspired O_2_ ≤4 L/min SpO_2_ >90% Inability to produce voluntary cough on command	Full ventilator support Upper airway obstruction confirmed by FOB Fully alert and producing voluntary cough on command Fenestrated TT in place
Marchese et al. [25]	Italy	2010	Retrospective questionnaire based Multicentre study (22 centres)	Acute respiratory failure, 24 COPD, 34 Neuromuscular diseases, 28 Surgical, 11 Thoracic dysmorphism, 4 OSAS, 2	719	50–78	Not mentioned. Majority patients with chronic diseases	Surgical, 34% PCT, 66%	NA	NA
Budviewser et al. [20]	Germany	2011	Retrospective single centre	AECOPD, 63 Pneumonia, 38 Cardiac failure, 18 Sepsis, 8 ARDS, 7	384	60–74	38	PCT, 100%	Tolerates TT capping >24–48 h Tracheostomy retainer (TR) successfully inserted ≥1h	NA
Shrestha KK et al. [9]	India	2012	Prospective single centre	Severe head trauma (GCS <8)	118		NA	NA	NA. Gradual vs. abrupt decannulation compared	NA
Warnecke T et al. [16]	Germany	2013	Prospective single centre	Neurologically ill patients, like stroke, ICH, GBS, meningoencephalitis	100		7–33	NA	Weaned off ventilator Assessment by CSE which includes: Patient’s vigilance and compliance, cough, swallowing assessed by fibreoptic endoscopic evaluation (FESS) with FEES protocol steps. Each step to be passed for decannulation to be considered, like secretions, spontaneous swallows, cough, puree consistency and fluids.	NA
Kenneth B et al. [21]	USA	2014	Retrospective single centre	Critically ill obese BMI 41.9 ± 14.3	102		NA	Surgical, 74% PCT, 26% Data missing—2	NA	Malignancy or tracheostomies performed outside
Pandain V et al. [17]	USA	2014	Prospective single centre	NA	57		21	NA	1.TT size ≤4 preferably cuffless 2. Breathes comfortably with continuous finger occlusion of TT >1 min without trapping air, tolerate speaking valve during waking hours without distress, mobilize secretions 3. Suction frequency less than every 4 h 4. No sedation during capping	Not satisfying inclusion criteria
Guerlain J et al. [18]	France	2015	Prospective single centre	Postoperative head and neck cancer patients	56		Short-term (<3 days)	Surgical, 100%	NA	NA
Pasqua et al. [22]	Italy	2015	Retrospective single centre	Respiratory (COPD, ILD, OSAS), 33 Cardiac, 10 Abdominal surgery, 4 Orthopaedic, 1	48		91.61–215.5	NA	Clinical and hemodynamic stability No evidence of sepsis Expiratory muscle strength (MEP >50 cm H_2_O) Absence of tracheal stenosis/granuloma Normal deglutition PaCO_2_ <50 mm Hg PaO_2_/FiO_2_ >200 Absence of nocturnal oxyhemoglobin desaturation Patient consent	NA
Cohen et al. [23]	Israel	2016	Retrospective single centre	Patients with ≥3 co-morbidities, 35%	49		10	PCT, 100%	Maturation of TT stoma Normal vital signs Effective coughing Normal swallowing Positive leak test	Age <18 years Complications during initial TT placement Decannulation process completed outside institute

**Table 2:** Characteristics of included studies

Author (Ref)	Method of decannulation	Primary outcome	Secondary outcome	Failure rate (%)	Time to recannulation	Limitations	Inference
Graves A et al. [11]	TT occlusion protocol after downsizing to fenestrated cuffed 7/8 portex tube	Decannulation	Decannulation	20	NA	NA	Even without FOB decannulation can be done with good success rate following long term MV
Bach et al. [12]	After measuring peak cough flow (PCF), switched to fenestrated cuffed TT that can be capped. Use of Nasal IPPV and MI–E, tube capped. If successful, TT removed, site closed, NIV and assisted coughing continued.	Decannulation	Factors predicting successful decannulation: Age Extent of pre-decannulation ventilator use Vital capacity Peak cough flow (PCF)	32	Within 3 days	Specific to neuromuscular and long-term MV pts NIV given to decannulated pts	Patients decannulated irrespective of their ventilator capacity. PCF >160 L/min predicted success Whereas <160 L/min predicted need to replace the tube
Ceriana et al. [8]	TT downsized to 6 mm and capped for 3–4 days Clinical stability Absence of psychiatric disorders Effective cough (MEP ≥40 cmH_2_O). PaCO_2_ <60 mmHg Adequate swallowing (Gag reflex or blue dye test) No tracheal stenosis endoscopically Spontaneous breathing for ≥5 days	Decannulation	NA	3.5	Up to 3 and 6 months	NA	Large majority of patients with clinical stability can be decannulated with reintubation rate less than 3% after 3 months
Leung et al. [19]	Not mentioned	Decannulation	Survival	6	During hospital stay.	Small sample size. Retrospective nature of the study.	ICU patients who require TT have high mortality (37%). All surviving patients were decannulated within 25 days. Patients with unstable or obstructed airway had shorter cannulation time compared to patients with chronic illness.
Tobin et al. [13]	Tolerate capping >24 h Cough effective (No need of suctioning) Speech (Passey–Muir valve)	Decannulation time from ICU discharge	LOS hospital LOS after discharge from ICU	13	NA	Retrospective data collection Lack of similar care in wards	Intensivist-led TT team is associated with shorter decannulation time and length of stay.
Stelfox et al. [24]	Tolerates TT capping (24 vs. 72 h) Effective cough (strong vs. weak) Secretions (thick vs. thin) Level of consciousness (alert vs. drowsy but arousable)	Which patient factors clinician’s rate as being important in the decision to decannulate? Which clinician and patient factors are associated with clinician’s recommendations to decannulate TT? Define decannulation failure. What do clinicians consider an acceptable rate of decannulation failure?	NA	20.4	Within 48 h (45% opinion) to 96 h (20% opinion) Acceptable rate of failure as 2–5%.	Only 73% responded to the questionnaire.	Patient’s level of consciousness, cough effectiveness, secretions, and oxygenation are all important determinants to decide decannulation.
Choate et al. [14]	Cuffless then check airflow through upper airway followed by TT removal	TD practice and failure rates during 4-year and 10-month study period	NA	5	Until discharge from hospital	Single centre study High % of trauma and neurosurgical patients Descriptive data Decannulation criteria not specified	Old age, prolonged duration of TT and retention of sputum were risk factors for failure
O Connor et al. [4]	TT occlusion with red cap/sleep apnea tube/Passy–Muir valve	Process of decannulation in patients of long-term acute care (LTAC) with prolonged MV (PMV)	NA	19	NA	Retrospective data collection	Decannulation was achieved in 35% of patients transferred to LTAC for weaning in patients with PMV
Chan LYY et al. [15]	Amount of TT secretions at different time intervals (4 times; 2 h apart) in the same day followed by induced peak cough flow rate (PCFR) by suction catheter	Decannulation	NA	6	Within 72 h	Air leakage during PCF rate estimation as most of them were on uncuffed TT Single centre Small sample	Induced PCF rate: 42.6 L/min in successful vs. 29 L/min in unsuccessful, where 29 L/min may be considered as the determinant point
Marchese et al. [25]	Scores for specific action Capping, 92/110 Tracheoscopy, 79/110 Tracheostomy button, 60/110 Downsizing, 44/110	Decannulation	Calculus score Each parameter score—0 to 5 (max score–110) 1: Difficult intubation 2: 1+ H/O Chronic respiratory failure 3: Home ventilation 4: 3+ ventilation hrs/day 5: PaCO_2_ in stable state 6: Impaired swallowing 7: Underlying disease 8: Cough effectiveness 9: Relapse rate last year	77	NA	NA	Substantial % maintained TT despite no requirement of MV No consensus on indications and systems for closure of TT
Budviewser et al. [20]	In patients with adequate cough and swallowing, the disc tracheostomy retainer (TR) is cut as per size of TT. Then inserted in a manner that it touches the ventral part of the trachea, thereby completely sealing the TT channel.	Decannulation	NA	*28*	Entire period of hospital stay	Did not measure PCF	Feasibility, efficacy and safety of TR in patients with prolonged weaning with high risk for recurrent or persistent hypercapnic respiratory failure
Shrestha KK et al. [9]	Abrupt: TT removal instantaneously. Gradual: Downsizing TT followed by strapping over the tube followed by strapping over the stoma. Gradual (68) vs. Abrupt (50)	Decannulation	Factors enhancing successful decannulation	Gradual (G)—1.5 Abrupt(A)—6 S (G)—98.5 S (A)—94	NA	NA	Factors associated with success were cough reflex, number of suctioning required per day, standard X-ray and use of antibiotics ≥7 days
Warnecke T et al. [16]	Clinical swallowing assessment (CSE) followed by fibreoptic endoscopic evaluation of swallowing (FEES) with decision to decannulate based only on FEES	Decannulation based on FEES	To compare how many could have been decannulated without FEES	1.9	Till discharge from hospital	Small % with neuromuscular weakness	FEES is an efficient, reliable, bedside tool, performed safely in tracheostomized critically ill neurologic patients to guide decannulation.
Kenneth B et al. [21]	Not mentioned	Tracheostomy type and patient outcome in terms of dependence, decannulation and death.	Patient factors associated with outcomes	49	NA	Retrospective data collection. Variability in co-morbidities(incomplete/incorrect medical records)	Increased tracheostomy dependence in OSA, and surgical tracheostomy
Pandain V et al. [17]	Capping	Quality improvement project to develop a standardized protocol for TT capping and decannulation process	NA	1.7	Tolerates capping 12–24 h No ↑ FiO_2_ >40%, shortness of breath, suction requirement, hemodynamic instability is defined as success	Small sample size Non-randomized Labour-intensive protocol	Multidisciplinary protocol for determining readiness to capping trial prior to decannulation
Guerlain J et al. [18]	Peak inspiratory flow (PIF) assessment through oral cavity after blocking TT cannula	Minimum peak inspiratory flow (PIF) required for successful decannulation	NA	13	Within 24 h	NA	PIF improves quality of care and optimizes outcomes following decannulation
Pasqua et al. [22]	Insertion of a fenestrated cannula in the TT followed by its closure with a cap for progressively longer periods up to 48 h	Evaluate efficacy of protocol to analyze factors that could predict successful decannulation	NA	37	NA	NA	Using specific protocol, decannulation can be done. However, larger prospective studies required.
Cohen et al. [23]	Study group: 3 step endoscopy Step 1—nasolaryngeal endoscopy confirming vocal cord mobility and normal supraglottis Step 2—TT removal Step 3—up and down look through TT stoma Control group: ↓TT or capping	Safety and feasibility of immediate decannulation compared to traditional decannulation	NA	20: control 0: study groups respectively		Single centre Retrospective analysis Clinical decisions based on single person opinion Potential bias	Immediate decannulation may be a safer alternative for weaning

*Abbreviations*: *NA* not available, *RR* respiratory rate, *SaO*_*2*_ arterial oxygen saturation, *TT* tracheostomy tube, *FOB* fibre optic bronchoscope, *MV* mechanical ventilation, *N* normal, *PaO*_*2*_ partial pressure of arterial oxygen, *IV* intravenous, *IPPV* intermittent positive pressure ventilation, *MI–E* mechanical insufflator–exsufflator, *NIV* non-invasive ventilation, *PCF* peak cough flow, *PIF* peak inspiratory flow, *MEP* maximum expiratory pressure, *PaCO*_*2*_ arterial partial pressure of carbondioxide, *LOS* length of stay, *ICU* intensive care unit, *AECOPD* acute exacerbation of chronic obstructive pulmonary disease, *PCT* percutaneous tracheostomy, *LTAC* long-term acute care, *PMV* prolonged mechanical ventilation, *ARDS* acute respiratory distress syndrome, *GCS* Glasgow coma scale, *ICH* intracranial haemorrhage, *GBS* Guillain–Barré syndrome, *CSE* clinical swallowing examination, *FESS* fibreoptic endoscopic evaluation of swallowing, *SCI* spinal cord injury, *TR* tracheostomy retainer, *OSA* obstructive sleep apnea syndrome, *ILD* interstitial lung disease, *FiO*_*2*_ fraction of inspired oxygen concentration

Majority of the studied tracheostomized patients had illnesses chronic in nature [8, 11]. The clinical spectrum included patients with stroke, quadriplegia, GBS, head trauma, acute exacerbations of chronic obstructive pulmonary disease, obstructive sleep apnea, restrictive lung disorder, acute respiratory distress syndrome, cardiac failure, cancer and postoperative neurosurgical, cardiothoracic and abdominal patients. The study by Kenneth B et al. specifically included critically ill obese patients with an average body mass index of 41.9 ± 14 [21]. Few studies [8, 13, 20, 23] reported the severity of the illness of included patients.

Ten out of 18 studies did not report whether the tracheostomy was performed by surgical or percutaneous technique. There were 2 studies each with tracheostomies performed by either surgical [8] or percutaneous [20] technique, while 5 studies included patients with both techniques [13, 14, 19, 21, 25].

The duration of MV prior to decannulation was quite variable. It was lesser than 3 days in the study by Guerlain J et al. [18] to as long as 2224 days with Bach et al. [12].

While the inclusion criteria were distinctly spelled out in 12 studies [8, 11–17, 20, 22], the exclusion criteria were only mentioned in 6 [14, 15, 17, 21, 23].

Readiness to decannulate was assessed by qualitative and quantitative determinants of coughing and swallowing in different studies. Peak cough flow (PCF) [20] and maximum expiratory pressure (MEP) [8] were used as quantitative measures of coughing. Swallowing was mostly assessed subjectively via gag reflex or dye test [2], except in the study by Wranecke et al. wherein fibreoptic endoscopic evaluation of swallowing (FEES) was used for objective assessment [23].

Specific method of decannulation was mentioned in all studies except two [19, 21]. Patients satisfying the criterion for decannulation were initially switched over to a smaller downsized fenestrated or non-fenestrated TT, which was later uncuffed and/or capped for a variable observation period before being finally removed. However, capping without downsizing [4, 13] and abrupt TT removal was also reported [9]. While spontaneous respiratory workload post downsizing TT was monitored in most studies, Bach et al. used NIV support to decrease the breathing workload [12].

While the primary outcome in most studies was a successful decannulation, the secondary outcomes were quite variable. These secondary outcomes included survival, length of stay, prediction factors for success, and utility of a particular assessment technique [16] or a screening tool [25]. In most studies, a successful decannulation occurred when there was no need of reinsertion of TT. However, the period of observation during which re-insertion was averted varied widely from a minimum of 24 h [18] to 3–6 months [8] and/or until discharge from the unit or hospital [14, 19]. The success rate of decannulation in the studies varied from as low of 23% [25] to as high as 100% [23].

The authors concluded from the studies that identification of patients ready for decannulation via objective assessment of swallowing (FEES) [16], coughing [PCF or peak [12, 18] inspiratory flow (PIF)] and use of a scoring (QsQ) system [26] performed by a multidisciplinary decannulation team in ICU may prove to be more successful.

According to the Q-Coh tool [10] majority of the studies were of low quality, except the study by Ceriana et al. [8], Chaote et al. [14] and Wranecke et al. [13]. Details of all attributes of the Q-Coh tool were as depicted in the Additional file 1: Table S1.

**Fig. 2:**
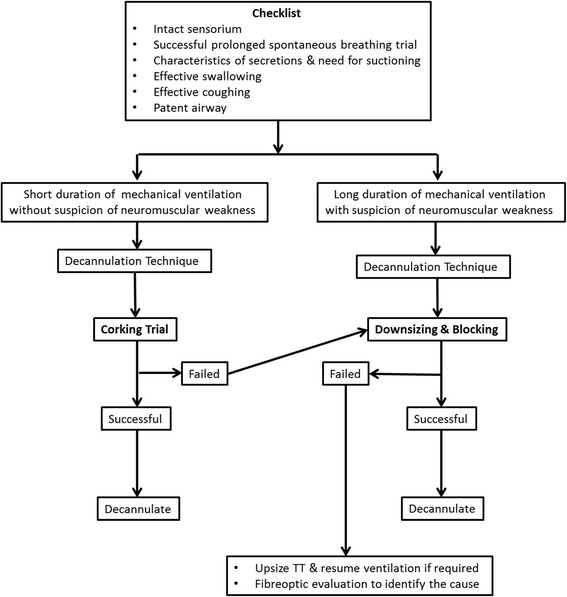
Decannulation algorithm

After this systematic review, we designed a protocolized bedside decannulation algorithm for use in our ICU (Fig. 2). This protocol is being currently studied in a prospective randomized manner to assess its feasibility in adult mechanically ventilated ICU patients.

## Discussion

Decannulation in tracheostomized patient is the final step towards liberation from MV. Despite its relevance, lack of a universally accepted protocol for decannulation continues to plague this vital transition. In order to focus attention on various practices of the process of tracheostomy decannulation, we decided to do this systematic review. The main finding from this review is that there is no randomized controlled study on this critical issue. Several individualized, non-comparative and non-validated decannulation protocols exist. However, a blinded randomized controlled study, either comparing protocolized and non-protocolized (usual practice) decannulation or comparing two different decannulation protocols, is urgently needed.

After ascertaining intactness of sensorium, further identification of patient’s readiness to decannulate is mostly based on the assessment of coughing and swallowing. More often than not these assessments are based on subjective clinical impression of the physician who may or may not be the most experienced one at the time of decannulation. This is an avoidable lacuna in care of tracheostomized patients. Busy units and busy physicians may devote minimal time for this transition. Protocolized decannulation in our opinion may guarantee consistency and objectivity of care.

As is obvious from the studies included in our systematic review, assessments were mostly subjective, although objective FEES [16] and of coughing with PCF [12] or PIF [18] have also been attempted. Endoscopic evaluation of swallowing though technically demanding provides an objective assessment. However, studies in support of this approach are limited. Only two studies [16, 23] out of 18 incorporated fibreoptic endoscopic evaluation of vocal cords and/or swallowing prior to decannulation. Warnecke T et al. in their study performed a mandatory step of FEES in their decannulation process [16]. In a recent retrospective study by Cohen et al., a three-step endoscopic confirmation of vocal cord mobility and normal supraglottis was ascertained prior to immediate decannulation [23]. He considered immediate decannulation as a safer and shorter alternative for weaning in tracheostomized patients as compared to traditional decannulation. When so many decannulations can happen without FEES, then what extra benefit does this technically demanding step offer over clinical swallowing evaluation (CSE) needs to be ascertained. Graves et al. [11] also concluded about good success rate without fibreoptic evaluation prior to decannulation of long-term MV patients. Availability and technical expertise of FEES needs to be ensured before including it in any decannulation protocol.

Similarly, subjective assessment of coughing is the usual norm. Only Bach et al. [12] in 1996, Ceriana et al. [8] in 2003, Chan LYY et al. [15] in 2010 and Guerlain J et al. [18] in 2015 used an objective measure of an effective cough to decide about decannulation. PCF, MEP and PIF are all parameters used by these investigators as measures of an effective cough. However, superiority of one over the other is undecided.

The adopted method of decannulation is also variable. While some authors preferred TT occlusion after downsizing to fenestrated or non-fenestrated tube [8, 11], others straight away capped the TT without downsizing [4, 13], while some abruptly removed the TT [9, 14]. The choice of the method is based on patient’s tolerability of the procedure and also on the physician’s experience. There exists no universally accepted method. Furthermore, discrepancy also exists in the period of observation before which decannulation is deemed successful. Probably, a combination of factors like the period of MV prior to decannulation, anticipation of neuromuscular fatigue on account of respiratory workload and protection of airway all play a role.

The self-confessed limitations of the included studies were as depicted in Table 2. Specific illness group, small sample size, retrospective design, and non-standardized, non-protocolized and non-validated method of decannulation are the major limitations of the included studies. But above all, absence of a randomized controlled study in this aspect of care is a major hurdle. The previously published systematic review on tracheostomy decannulation was by Santus P et al. [26] in 2014. Our systematic review has included 10 of these studies apart from addition of another 8. While he compared primary and secondary outcomes of included studies, our review is much more exhaustive in that it incorporates the relevant details of 18 studies in a concise tabular form. Our systematic review also incorporates the Q-Coh tool [10] to assess the methodological quality of included cohort studies. As none of the studies included are of desired quality, the need for randomized controlled study on decannulation cannot be over emphasized. However, our systematic review also has several limitations. We have not searched other databases like Google Scholar, Scopus or EMBASE and also not included non-English language articles.

Our protocolized decannulation algorithm (Fig. 2) incorporates easy to use bed-side checklist for evaluation of patients deemed fit for decannulation. The screening checklist includes assessment for intactness of sensorium, characteristics of secretions and need and frequency of suctioning, effectiveness of swallowing and coughing, patency of airway and successfulness of a prolonged spontaneous breathing trial (SBT). The patient should be conscious, oriented and be able to maintain a patent airway. Secretions should be easy to handle by the patient and frequency of suctioning should be less than 4 in the previous 24 hours. The patient must be able to swallow liquids/semisolids without risk of aspiration, have adequate cough with good peak expiratory flow rate (PEFR) (>160 L/min) and be able to maintain a patent airway. Patency of the airway can be assessed bedside by simply deflating the cuff and occluding the TT with a gloved finger for testing phonation of the patient. In patients with prolonged MV of greater than 4 weeks, the duration of successful SBT should preferably be 48 hours or more. After the initial screening checklist, decision about the decannulation technique is based on the duration of MV and presence of neuromuscular weakness. Patients with less than 4 weeks of MV and with no suspicion of neuromuscular weakness are subjected to a corking trial. This trial involves blocking the existing TT after cuff deflation followed by careful instructions to the bedside nurse/physician to re-inflate the cuff in case of respiratory distress. Depending on the tolerability and absence of any distress the TT is decannulated. However, in case of a failed corking trial the TT can be downsized and blocked followed by a period of careful observation for few hours. If the observation period is not associated with any respiratory distress decannulation can then be performed. Patients who failed the corking trail as well as downsizing & blocking and are in respiratory distress need immediate upsizing of the TT to resume ventilation. Further assessment warrants a FOB examination to explore the cause of failure. In patients with MV for more than 4 weeks and with suspicion of neuromuscular weakness the decannulation technique is that of downsizing and blocking. In case of failure and respiratory distress, approach remains same as above. This protocol is currently under evaluation in our unit via a randomized study.

## Conclusions

Decannulation is an essential step towards liberating a tracheostomized patient from mechanical ventilation. This transition is more often individualized than protocolized. Universally accepted protocol is needed for better standardization. Randomized controlled studies in this aspect of tracheostomy care can make it more evidence based.

## Additional file

Additional file 1: Table S1. Quality of cohort studies as assessed by Q-Coh tool. (DOCX 26 kb)

## Abbreviations

CSE: Clinical swallowing examination
FESS: Fibreoptic endoscopic evaluation of swallowing
HDU: High dependency unit
ICU: Intensive care unit
MEP: Maximum expiratory pressure
MV: Mechanical ventilation
NIV: Non-invasive ventilation
PCDT: Percutaneous dilatational tracheostomy
PCF: Peak cough flow
PIF: Peak inspiratory flow
TT: Tracheostomy tube
