# Supplementary material for: The practice of tracheostomy decannulation—a systematic review
Source: J Intensive Care. 2017 Jun 20;5:38. doi: 10.1186/s40560-017-0234-z (PMC5477679; doi:10.1186/s40560-017-0234-z)
Supplement: Additional file 1: Table S1. — Quality of cohort studies as assessed by Q-Coh tool. (DOCX 26 kb) [file 40560_2017_234_MOESM1_ESM.docx]

**Table S1: Quality of Cohort studies as assessed by Q-Coh Tool**

| **CHARACTERISTICS**  **OF THE STUDY** | Graves A, et al ^11^ | Bach, et al ^12^ | Ceriana, et al^l 8^ | Leung, et al^19^ | Tobin, et al^13^ | Stelfox, et al^24^ | Choate, et al^14^ | O Connor , et al^4^ | Chann LYY , et al ^15^ | Marchese,  et al^25^ | Budviewser, et al ^20^ | Shrestha KK , et al ^9^ | Warnecke T, et al ^16^ | Kenneth B , et al ^21^ | Pandian V , et al ^17^ | Guerlain J , et al ^18^ | Pasqua, et al^22^ | Cohen , et al^23^ |
| --- | --- | --- | --- | --- | --- | --- | --- | --- | --- | --- | --- | --- | --- | --- | --- | --- | --- | --- |
| **DESIGN** | | | | | | | | | | | | | | | | | | |
| Is there a comparison between at least two groups to assess the effect/ association of an exposure and an outcome? (Yes/No) | N | N | N | N | N | N | N | N | N | N | N | Y | N | N | N | N | N | N |
| Are the groups defined by the exposure variable? (Yes/No) | NA | NA | NA | NA | NA | NA | NA | NA | NA | NA | NA | Y | N | N | N | N | NA | NA |
| Has or could any of the participants have the outcome of interest on onset? | N | N | N | N | N | N | N | N | N | N | N | N | N | N | N | N | N | N |
| Do investigators handle who is exposed or not? (No/Yes) | Y | Y | Y | Y | N | N | N | N | N | N | N | N | N | N | N | N | N | N |
| Is information about the exposure and the outcome of interest registered concurrently? (No/Yes) | N | N | Y | N | N | N | N | N | N | N | N | Y | N | N | N | N | N | N |
| **INFERENCE: Is the tool suitable for this study? (Yes/No)** | ***N*** | ***N*** | ***N*** | ***N*** | ***N*** | ***N*** | ***N*** | ***N*** | ***N*** | ***N*** | ***N*** | ***N*** | ***N*** | ***N*** | ***N*** | ***N*** | ***N*** | ***N*** |
| **REPRESENTATIVENESS** | | | | | | | | | | | | | | | | | | |
| Have the study participants been selected using a randomized sampling procedure? (Yes/No) | N | N | N | N | N | N | N | N | N | N | N | N | N | N | N | N | N | N |
| Is the similarity between the selected group of subjects and the target population justified by the authors? (Yes,  empirically/Yes, verbally/No) | N | N | N | N | N | N | N | N | N | N | N | N | N | N | N | N | N | N |
| Is there a predominant reason for refusing to participate at the beginning of the study? (No-Irrelevant/Yes/Not  reported) | NI | NI | NI | NI | NI | NI | NI | NI | NI | NI | NI | NI | NI | NI | NI | NI | NI | NI |
| **INFERENCE:** Could the results be generalized from the sample to the target population? (Probably/Unlikely) | ***U*** | ***U*** | ***U*** | ***U*** | ***U*** | ***U*** | ***U*** | ***U*** | ***U*** | ***U*** | ***U*** | ***U*** | ***U*** | ***U*** | ***U*** | ***U*** | ***U*** | ***U*** |
| **COMPARABILITY OF GROUPS** | | | | | | | | | | | | | | | | | | |
| Were the inclusion and exclusion criteria explicitly defined for all groups? (Yes/No) | N | N | Y | N | N | N | Y | N | Y | N | N | N | N | N | Y | N | N | Y |
| Were the same inclusion and/or exclusion criteria applied equally to all groups? (Yes/No/Not Reported) | Y | NR | Y | NR | N | NR | NR | N | Y | N | N | N | N | N | Y | N | N | N |
| Could differences in the selection criteria introduce systematic differences between the groups (other than exposure)? (Unlikely/Probably) | P | P | U | P | U | P | P | P | P | P | P | P | P | P | P | P | P | P |
| Were known confounding factors accounted for in the design or in the analysis? (Yes/Partially/No) | Y | Y | P | Y | N | Y | Y | N | P | N | N | Y | Y | Y | Y | Y | N | N |
| **INFERENCE:** Is bias between the groups avoided at the beginning of the study? (Probably/Unlikely) | ***U*** | ***U*** | ***U*** | ***U*** | ***U*** | ***U*** | ***U*** | ***U*** |  | ***U*** | ***U*** | ***U*** | ***U*** | ***U*** | ***U*** | ***U*** | ***U*** | ***U*** |
| **EXPOSURE MEASURE** | | | | | | | | | | | | | | | | | | |
| Was the exposure explicitly defined? (Yes/No) | N | N | N | N | N | N | N | N | N | N | N | N | N | N | N | N | D | N |
| Was the tool used to measure the exposure variable valid? (Yes/Presumably/Doubtfully) | D | D | Pr | D | D | D | D | D | D | D | D | D | D | D | D | D | D | D |
| Was the tool used to measure the exposure variable reliable? (Yes/Presumably/Doubtfully) | P | D | Pr | D | D | D | D | D | D | D | D | D | D | D | D | D | D | D |
| Was the procedure to measure the exposure the same for all participants? (Yes/No/Not Reported) | Y | N | Y | NR | NR | NR | NR | NR | NR | NR | NR | NR | NR | NR | NR | NR | NR | NR |
| **INFERENCE:** Could the classification of the participants into exposed or unexposed be biased? (Unlikely/Probably) | ***NA*** | ***NA*** | ***U*** | ***P*** | ***P*** | ***P*** | ***P*** | ***P*** | ***P*** | ***P*** | ***P*** | ***P*** | ***P*** | ***P*** | ***P*** | ***P*** | ***P*** | ***P*** |
| **MAINTENANCE OF COMPARABILITY** | | | | | | | | | | | | | | | | | | |
| Were potential confounders that appeared during the follow-up time taken into account in the analyses? (Yes/No) | N | N | N | Y | N | Y | N | N | N | N | N | N | N | N | N | N | N | N |
| Was the length of follow-up similar between the groups? (Yes/No, but controlled/No) | Y | N | Y | N | N | N | N | N | N | N | N | N | N | N | N | N | N | N |
| Is there any potential confounder that could have appeared during follow-up that was not taken into account by the authors? (Probably none important/Probably/Yes) | PY | Y | N | P | Y | Y | Y | Y | Y | Y | Y | Y | Y | Y | Y | Y | Y | Y |
| **INFERENCE:** Could the exposure to other factors appearing during follow-up introduce systematic differences between the groups? (Unlikely/Probably) | ***U*** | ***P*** | ***U*** | ***P*** | ***P*** | ***P*** | ***P*** | ***P*** | ***P*** | ***P*** | ***P*** | ***P*** | ***P*** | ***P*** | ***P*** | ***P*** | ***P*** | ***P*** |
| **OUTCOME MEASURE** | | | | | | | | | | | | | | | | | | |
| Was the outcome variable explicitly defined? (Yes/No) | Y | N | Y | N | N | N | N | N | Y | N | Y | Y | N | N | N | N | Y | Y |
| Was the tool used to assess the outcome variable valid? (Yes/Presumably/No) | N | N | P | N | N | N | N | N | N | N | N | N | N | N | N | N | N | N |
| Was the tool used to assess the outcome variable reliable? (Yes/Presumably/No) | P | N | P | N | N | N | N | N | N | N | N | P | N | N | N | N | N | N |
| Was the tool used to assess the outcome appropriate? (Probably/Unlikely) | P | U | P | U | U | U | U | U | U | U | U | U | U | U | U | U | N | N |
| Was the outcome variable assessed in the same way in all groups? (Yes/No) | NA | N | NA | N | N | N | N | N | N | N | N | N | N | N | N | N | N | N |
| Was the outcome variable assessed at the same time for all groups? (Yes/No) | NA | N | N | N | N | N | N | N | N | N | N | N | N | N | N | N | N | N |
| Was the outcome variable assessed in the same context for all groups? (Yes/No) | NA | N | Y | N | N | N | N | N | N | N | N | N | N | N | N | N | N | N |
| Could the procedures for measuring the outcome variable introduce systematic differences between the groups?(Unlikely/Probably) | NA | P | P | P | P | P | P | P | P | P | P | P | P | P | P | P | N | N |
| Were the participants successfully blinded to the research question? (Yes/No/Not necessary) | N | NN | NN | NN | NN | NN | NN | NN | NN | NN | NN | NN | NN | NN | NN | NN | NN | NN |
| Were those assessing the outcome successfully blinded to the exposure status of the participants?  (Yes/No/Not necessary) | N | NN | NN | NN | NN | NN | NN | NN | NN | NN | NN | NN | NN | NN | NN | NN | NN | NN |
| **INFERENCE:** Does the measure of the outcome variable reflect the true situation? (Probably/Unlikely) | ***U*** | ***U*** | ***P*** | ***U*** | ***U*** | ***U*** | ***U*** | ***U*** | ***U*** | ***U*** | ***U*** | ***U*** | ***U*** | ***U*** | ***U*** | ***U*** | ***U*** | ***U*** |
| **ATTRITION** | | | | | | | | | | | | | | | | | | |
| Were dropout rates similar in all groups? (Yes/No/Not Reported) | NR | NA | NA | NR | N | NR | N | N | N | N | N | N | N | N | N | N | N | N |
| Were reasons for dropping out similar in all groups? (Yes/No/Not Reported) | NR | NA | NA | NR | NR | NR | N | N | N | N | N | N | N | N | N | N | N | N |
| **INFERENCE:** Could incomplete information introduce systematic differences between groups? (Unlikely/Probably) | ***U*** | ***P*** | ***U*** | ***P*** | ***P*** | ***P*** | ***U*** | ***U*** | ***U*** | ***U*** | ***U*** | ***U*** | ***P*** | ***U*** | ***U*** | ***U*** | ***U*** | ***U*** |
| **STATISTICAL ANALYSIS** | | | | | | | | | | | | | | | | | | |
| **INFERENCE:** *Do the results of the statistical analysis reflect the true situation? (Probably/Unlikely)* | ***U*** | ***U*** | ***P*** | ***U*** | ***U*** | ***U*** | ***P*** | ***U*** | ***U*** | ***U*** | ***U*** | ***U*** | ***P*** | ***U*** | ***U*** | ***U*** | ***U*** | ***U*** |
| **OVERALL ASSESSMENT OF THE STUDY’S QUALITY** | | | | | | | | | | | | | | | | | | |
| *What overall quality does this study have?*  *(Good /Acceptable /Low)* | ***L*** | ***L*** | ***A*** | ***L*** | ***L*** | ***L*** | ***A*** | ***L*** | ***L*** | ***L*** | ***L*** | ***L*** | ***A*** | ***L*** | ***L*** | ***L*** | ***L*** | ***L*** |

*Abbreviations: Y, yes; N, no; P, probably; Pr, presumably; U, unlikely; NA, not available; NR, not reported; NN, not necessary; NI, no irrelevant; L, low; D, doubtfully; A. Acceptable.*
